# Peripartum Depression as a Heart–Brain–Endocrine–Immune Syndrome: Neuroendocrine, Cardiovascular, and Inflammatory Pathways Underlying Maternal Vulnerability

**DOI:** 10.3390/life16020236

**Published:** 2026-02-01

**Authors:** Giuseppe Marano, Marianna Mazza

**Affiliations:** 1 Department of Neuroscience, Head-Neck and Chest, Section of Psychiatry, Fondazione Policlinico Universitario Agostino Gemelli IRCCS, Largo Agostino Gemelli 8, 00168 Rome, Italy; 2 Department of Neuroscience, Section of Psychiatry, Università Cattolica del Sacro Cuore, 00168 Rome, Italy

**Keywords:** autonomic dysfunction, biomarkers, endothelial dysfunction, heart–brain axis, inflammation, neuroendocrine dysregulation, peripartum depression, precision psychiatry

## Abstract

Peripartum depression (PPD) represents one of the most prevalent and disabling psychiatric conditions among women, yet its underlying biology remains poorly integrated across medical disciplines. Emerging evidence highlights PPD as a prototypical disorder of the heart–brain axis, where neuroendocrine changes, immune activation, and cardiovascular dysregulation converge to shape maternal vulnerability. During pregnancy and the postpartum period, abrupt fluctuations in estrogen, progesterone (P4), and placental corticotropin-releasing hormone (CRH) interact with a sensitized hypothalamic–pituitary–adrenal (HPA) axis, altering neural circuits involved in mood regulation, stress reactivity, and maternal behavior. Parallel cardiovascular adaptations, including endothelial dysfunction, altered blood pressure variability, and reduced heart rate variability (HRV), suggest a profound perturbation of autonomic balance with potential long-term implications for maternal cardiovascular health. Neuroinflammation, microglial activation, and systemic cytokine release further mediate the bidirectional communication between the heart and the brain, linking emotional dysregulation with vascular and autonomic instability. Evidence also indicates that conditions such as preeclampsia and peripartum cardiomyopathy share biological pathways with PPD, reinforcing the concept of a unified pathophysiological axis. This review synthesizes current knowledge on the neurobiological, cardiovascular, endocrine, and inflammatory mechanisms connecting PPD to maternal heart–brain health, while discussing emerging biomarkers and therapeutic strategies aimed at restoring integrative physiology. Understanding PPD as a multisystem heart–brain disorder offers a transformative perspective for early detection, risk stratification, and personalized intervention during one of the most biologically vulnerable periods of a woman’s life.

## 1. Introduction

Peripartum depression (PPD), encompassing major depressive episodes occurring during pregnancy or within the first 12 months postpartum, represents one of the most prevalent and impactful psychiatric conditions affecting women worldwide. Current estimates suggest that 10–20% of women develop clinically significant depressive symptoms during the peripartum period, with higher rates among those exposed to psychosocial stressors, obstetric complications, or a prior history of affective disorders [1,2]. Beyond its immediate psychological burden, PPD exerts profound consequences on maternal morbidity, infant neurodevelopment, and long-term family well-being, constituting a major public health issue with wide-reaching implications [3,4].

Despite extensive research, the underlying biological mechanisms of PPD remain insufficiently integrated across medical disciplines. Traditional conceptualizations framed PPD primarily as a psychiatric response to psychosocial adversity or hormonal withdrawal; however, this reductionistic perspective fails to capture the complex, multisystemic physiological transitions that characterize pregnancy and the postpartum period. In recent years, a growing body of evidence has pointed toward a more nuanced pathophysiological landscape, wherein neuroendocrine, cardiovascular, immune, and autonomic systems interact dynamically to shape maternal vulnerability to affective dysregulation [5,6]. This perspective has given rise to the notion of PPD as a prototypical heart–brain syndrome: a condition emerging from dysregulated communication between the central nervous system and peripheral physiological systems, particularly the cardiovascular and immune axes.

Several theoretical frameworks have contributed to the current understanding of peripartum depression by focusing on specific domains such as psychosocial stress, reproductive hormone fluctuations, hypothalamic–pituitary–adrenal (HPA) axis dysregulation, or immune activation. These models have provided relevant insights into discrete mechanisms of vulnerability, yet they often consider biological systems in relative isolation or emphasize unidirectional pathways. An integrative perspective that accounts for the dynamic and reciprocal interactions among neuroendocrine, autonomic, cardiovascular, and immune processes across pregnancy and the postpartum period remains less fully developed. Situating peripartum depression within such a multisystem physiological context allows for a more comprehensive interpretation of maternal vulnerability, one that reflects the complexity of the peripartum transition and its impact on both mental and somatic health.

While previous reviews have addressed individual systems such as neuroendocrine, immune, or autonomic pathways in PPD, the present review is novel in three complementary ways: it emphasizes the cardiovascular and autonomic contributions to PPD, highlights the bidirectional interactions within the heart–brain–immune network, and integrates these pathways into a unified clinical framework that may inform risk stratification and therapeutic strategies.

During pregnancy, the maternal organism undergoes one of the most profound endocrine recalibrations of the human lifespan. Levels of estrogen, progesterone (P4), and their neuroactive metabolites, such as allopregnanolone (ALLO), rise exponentially, modulating synaptic plasticity, GABAergic tone, and stress reactivity [7]. Simultaneously, placental corticotropin-releasing hormone (CRH) increases dramatically, reshaping the hypothalamic–pituitary–adrenal (HPA) axis set point. Following delivery, the precipitous decline of these hormones interacts with changes in glucocorticoid sensitivity, rendering certain women particularly susceptible to affective instability. Notably, the magnitude and direction of HPA axis changes differ among women who develop PPD, suggesting individual variability in endocrine sensitivity and stress pathway recalibration [8].

In parallel with these endocrine transformations, pregnancy demands extensive cardiovascular adaptation to support fetal growth and maternal homeostasis. Cardiac output increases by up to 50%, systemic vascular resistance decreases, and the autonomic nervous system (ANS) adjusts to maintain hemodynamic stability. In susceptible individuals, these adaptations may fail to recalibrate efficiently postpartum, resulting in altered heart rate variability (HRV), increased blood pressure variability, and changes in endothelial function, biomarkers increasingly associated with mood disorders, including PPD [9]. Importantly, cardiovascular dysregulation in the early postpartum period has been linked to later-life cardiovascular disease, a risk amplified among women with a history of PPD [10]. This convergence of cardiovascular and affective risk underscores the need to conceptualize PPD as embedded within a broader maternal heart–brain physiology [4].

Beyond endocrine and cardiovascular factors, the maternal immune system undergoes major shifts across gestation and the postpartum period. Pregnancy is characterized by a finely calibrated state of immunological plasticity, alternating between pro-inflammatory and anti-inflammatory phases. Disruptions to this immune choreography, including microglial priming, increased circulating cytokines (e.g., interleukin-6 [IL-6], tumor necrosis factor-alpha [TNF-α], interleukin-1 beta [IL-1β]), and heightened inflammatory signaling, have been consistently linked to depressive symptoms postpartum [11,12]. Furthermore, inflammation acts not only as a risk mediator but also as an upstream regulator of endothelial function and HPA axis responsivity, highlighting its role as a central integrator within the heart–brain framework.

Crucially, several obstetric and cardiac conditions share biological pathways with PPD. Preeclampsia, for instance, involves endothelial dysfunction, oxidative stress, and exaggerated inflammatory activation, mechanisms also implicated in PPD. Similarly, peripartum cardiomyopathy (PPCM) has been associated with dysregulated prolactin cleavage, angiogenic imbalance, and systemic inflammation, all of which bear conceptual overlap with heart–brain disruption in PPD [13]. These convergences suggest that PPD emerges not from isolated neurochemical events but from complex dysregulations across neuroendocrine, cardiovascular, and immune networks, reinforcing the idea of a unified pathophysiological axis.

Recognizing PPD as a heart–brain syndrome offers several advantages. First, it reframes maternal mental health as inseparable from systemic physiology, bridging psychiatry, cardiology, endocrinology, and obstetrics. Second, it highlights novel biomarkers, such as HRV indices, placental CRH, angiogenic factors, and cytokine profiles, that may improve risk stratification and early detection. Third, it opens the door to innovative therapeutic approaches targeting systemic dysregulation, including neurosteroid modulation, anti-inflammatory interventions, autonomic rehabilitation, and cardio-protective strategies.

**Figure 1:**
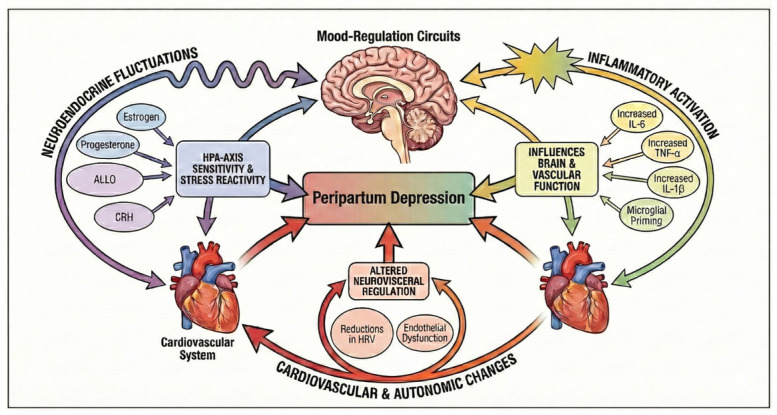
Multisystemic interactions linking the brain, endocrine, cardiovascular, and immune systems in peripartum depression. Note: The figure depicts the interconnected biological pathways that contribute to maternal vulnerability during the peripartum period. Neuroendocrine fluctuations (estrogen, progesterone, ALLO, corticotropin-releasing hormone) modulate HPA-axis sensitivity and stress reactivity. Cardiovascular and autonomic changes, such as reductions in HRV and endothelial dysfunction, reflect altered neurovisceral regulation. Immune activation (increased IL-6, TNF-α, IL-1β; microglial priming) further influences both brain and vascular function. Together, these systems converge on central mood-regulation circuits, supporting the conceptualization of PPD as a disorder of impaired heart–brain integration. Abbreviations. CRH: Corticotropin-Releasing Hormone.

This review synthesizes current advances across neuroendocrine, cardiovascular, autonomic, and inflammatory research to present an integrated conceptualization of PPD as a disorder of heart–brain communication. By mapping convergent biological pathways, we aim to elucidate mechanisms of maternal vulnerability, highlight emerging biomarkers with translational potential, and discuss therapeutic strategies capable of restoring heart–brain integration. Understanding PPD through this systemic lens may not only refine clinical practice but also support more personalized approaches to maternal mental health during one of the most physiologically vulnerable periods of life. Figure 1 provides a conceptual overview of the multisystemic heart–brain pathways implicated in peripartum depression. The figure illustrates how neuroendocrine fluctuations, autonomic and cardiovascular adaptations, and immune–inflammatory signals converge on central mood-regulation circuits, shaping maternal vulnerability.

## 2. Materials and Methods

This narrative review was conducted following standard methodological principles for non-systematic biomedical reviews. A comprehensive search strategy was applied to identify relevant studies examining the neuroendocrine, cardiovascular, autonomic, and immune mechanisms involved in PPD, as well as heart–brain interactions in pregnancy and the postpartum period.

A literature search was performed in PubMed/MEDLINE, Scopus, and Web of Science from inception to December 2024. Additional articles were identified through manual searches of reference lists and through targeted queries in Google Scholar. The following keywords and Boolean operators were used in various combinations: “peripartum depression” OR “postpartum depression” OR “maternal depression”; “heart–brain axis” OR “neurovisceral integration” OR “autonomic nervous system”; “neuroendocrine” OR “HPA axis” OR “CRH” OR “allopregnanolone”; “cardiovascular” OR “heart rate variability” OR “endothelial dysfunction”; “immune activation” OR “inflammation” OR “cytokines” OR “microglia”; “preeclampsia” OR “peripartum cardiomyopathy”. Search strings were adapted for each database to maximize coverage.

To ensure methodological rigor, titles and abstracts of all retrieved articles were independently screened by the two authors, and full texts were subsequently assessed for eligibility according to predefined inclusion and exclusion criteria. Discrepancies were resolved through discussion to reach consensus.

The review included: original research articles, reviews, meta-analyses, clinical guidelines, and experimental studies relevant to PPD and its biological mechanisms; studies published in English; research addressing at least one of the domains of interest: neuroendocrine regulation, cardiovascular/autonomic function, immune–inflammatory pathways, central neural circuits, or the integration across systems. Exclusion criteria included: conference abstracts without full data, case reports unless of exceptional mechanistic relevance, non-peer-reviewed material, and studies not addressing biological pathways.

**Figure 2:**
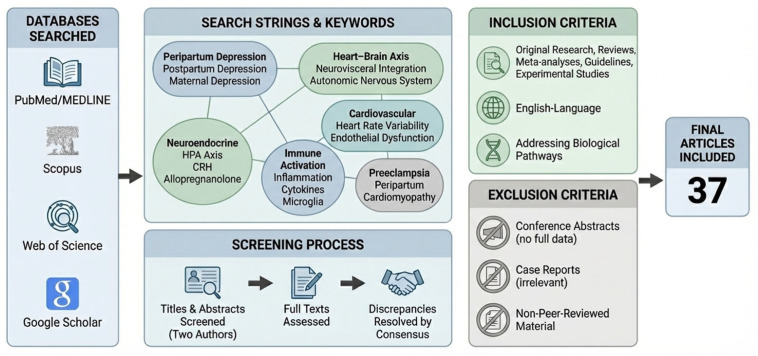
Flow-style schematic of literature identification and selection process for the narrative review.

Following application of these criteria, a total of 37 articles were identified and included in this review. To enhance transparency of the literature selection process,, a flow-style schematic summarizing the study identification and selection process is included to facilitate readability and methodological clarity (Figure 2).

### 2.1. Neuroendocrine Dysregulation in Peripartum Depression

Neuroendocrine fluctuations represent one of the most distinctive and dynamically regulated biological processes of the peripartum period. Pregnancy, childbirth, and the postpartum involve dramatic shifts in reproductive steroids, placental hormones, stress-related peptides, and neuroactive metabolites. These changes profoundly influence neural circuits involved in mood regulation, social bonding, and stress responsivity. Evidence accumulated in the past two decades has moved the field away from simplistic “hormone withdrawal” explanations toward a more integrative view in which individual sensitivity to endocrine change is central to the pathogenesis of PPD [7,14].

Rather than being triggered by absolute hormone levels, PPD may emerge when neurobiological systems fail to adapt to rapid endocrine transitions. This vulnerability interacts with stress, inflammation, and genetic predispositions, suggesting that neuroendocrine dysregulation could be an important factor within a multisystemic heart–brain framework.

### 2.2. Reproductive Steroids and Mood Regulation

Estradiol (E2) levels increase exponentially across pregnancy and then fall sharply after delivery, representing one of the most abrupt endocrine transitions in human physiology. Estrogens modulate neurotransmission in several mood-relevant circuits, including the serotonergic system through increased tryptophan hydroxylase expression, the dopaminergic system through modulation of mesolimbic reward pathways, and glutamatergic plasticity in hippocampal and prefrontal regions [15,16].

It has been observed that women with a history of reproductive mood disorders may show depressive symptoms when exposed to controlled estrogen and P4 withdrawal, whereas healthy controls typically remain asymptomatic [17]. This supports the concept of steroid sensitivity, which is now frequently considered in relation to PPD. Neuroimaging research confirms that women vulnerable to PPD exhibit increased amygdala reactivity and reduced prefrontal regulation during periods of estrogen fluctuation [18]. These findings indicate that the postpartum period may reveal individual differences in how the brain responds to changes in estrogen levels.

P4 undergoes a trajectory parallel to E2, peaking in late pregnancy and collapsing postpartum. Its metabolite ALLO powerfully enhances GABA-A receptor activity, exerting anxiolytic and stress-buffering properties [19]. Clinical studies indicate that lower late-pregnancy ALLO levels are associated with the onset of PPD, suggesting that altered GABAergic adaptation may contribute to increased susceptibility to mood destabilization [20,21].

Postmortem and preclinical studies demonstrete that chronic endocrine fluctuations alter GABA-A receptor subunit composition, transiently reducing benzodiazepine sensitivity and altering inhibitory control [22]. These findings offer a potential neurobiological explanation for why the postpartum period, characterized by rapid shifts in neurosteroids, may be associated with temporary alterations in inhibitory signaling.

The therapeutic success of brexanolone (intravenous ALLO) and zuranolone (oral analog), both restoring GABAergic modulation, is consistent with the hypothesis that neurosteroid deficiency may contribute to PPD in a subset of cases.

### 2.3. Placental CRH and the Stress Axis

Placental corticotropin-releasing hormone (pCRH) is a unique peptide hormone produced exclusively by the placenta, rising dramatically during the second and third trimesters. Unlike hypothalamic CRH, pCRH secretion is stimulated by cortisol, creating a feed-forward loop that accelerates as pregnancy progresses [23].

Elevated or rapidly rising pCRH levels have been associated with increased maternal cortisol reactivity [24], preterm birth and obstetric complications [25], and a higher likelihood of postpartum depressive symptoms [26,27]. These observations are consistent with the possibility that exaggerated pCRH trajectories may reflect a dysregulated stress axis and could contribute to challenges in postpartum HPA-axis recalibration.

Pregnancy is characterized by naturally elevated cortisol levels, blunted diurnal variation, and reduced glucocorticoid receptor responsiveness. After childbirth, a rapid normalization is expected; however, women who develop PPD frequently exhibit higher evening cortisol, altered cortisol awakening response (CAR), impaired negative feedback sensitivity, and dysregulated diurnal rhythms [28,29]. These abnormalities resemble patterns observed in stress-related disorders, suggesting a potential shared vulnerability phenotype linked to impaired endocrine–autonomic integration. Notably, disruptions in HPA-axis resetting have been associated with reduced vagal tone (HRV measures), further linking stress physiology with cardiovascular dysregulation in PPD.

### 2.4. Oxytocin, Bonding, and Maternal Behavior

Oxytocin is central to labor, lactation, stress attenuation, and mother–infant bonding. Several longitudinal studies indicate that women with lower gestational or early postpartum oxytocin levels may be more likely to experience depressive symptoms and bonding difficulties [30,31].

Mechanistically, oxytocin influences amygdala threat reactivity, ventral striatum reward responses to infant cues, hypothalamic autonomic output, and stress-related HPA modulation. Women with PPD have been reported to show lower oxytocin release during breastfeeding, altered oxytocin receptor (OXTR) methylation, and impaired reward processing of infant faces, suggesting potential hormonal and epigenetic vulnerability [32,33].

### 2.5. Thyroid Function and Peripartum Mood Disturbance

The peripartum period imposes high metabolic demands on the thyroid gland. Subclinical hypothyroxinemia, elevated thyroid peroxidase antibodies, and postpartum thyroiditis have been linked to depressive symptoms in some studies, even among euthyroid women [34,35].

Thyroid hormones regulate serotonergic tone, mitochondrial function, and hippocampal neurogenesis. Although thyroid dysfunction is not universally present in PPD, it may act as a biological factor that amplifies vulnerability in individuals with pre-existing stress or neuroendocrine dysregulation.

### 2.6. Brain Circuitry and Endocrine Sensitivity

Neuroendocrine fluctuations modulate neural circuits implicated in maternal adaptation. These include: amygdala, showing heightened reactivity under low ALLO or low oxytocin states; prefrontal cortex, reducing regulatory control during estrogen withdrawal; hippocampus, vulnerable to elevated cortisol and inflammation; ventral striatum, showing reduced reward responsiveness in PPD [36].

These circuit-level perturbations may help explain why endocrine transitions are associated with affective symptoms primarily in vulnerable individuals.

A key conceptual shift is the recognition that PPD may not be solely determined by hormonal levels but also by the brain’s adaptive capacity to respond to hormonal transitions [14]. Endocrine sensitivity is influenced by factors such as genetic variation in estrogen/P4 receptors [37], early-life stress altering HPA and GABAergic systems [38], inflammatory signals modulating steroid receptors [39], epigenetic marks on hormone-responsive genes [33].

This model explains why most women tolerate dramatic hormonal shifts without developing mood disorders, whereas a subset develops severe symptoms even with normal endocrine profiles. The neuroendocrine model of PPD has important translational implications. Therapeutic agents such as brexanolone and zuranolone support the potential role of neurosteroid withdrawal and GABAergic instability [40]; oxytocin pathways may represent targets for future interventions addressing bonding difficulties and stress dysregulation; CRH and cortisol trajectories are being explored as early biomarkers of vulnerability; and lifestyle interventions that modulate stress physiology (HRV biofeedback, mindfulness) may aid HPA recalibration postpartum. Given the complexity of endocrine fluctuations across pregnancy and postpartum, Table 1 organizes the most relevant biomarkers into functional domains, highlighting how alterations in neurosteroids, HPA-axis dynamics, and oxytocin signaling contribute to PPD pathophysiology.

**Table 1:** Key Neuroendocrine Biomarkers Implicated in Peripartum Depression (PPD).

Biomarker	Physiological Role	Pattern Associated with PPD Risk	Mechanistic Implications	Key References
Allopregnanolone (ALLO)	Enhances GABAergic inhibition; reduces stress	Lower late-pregnancy or postpartum levels	Reduced GABA-A R modulation → heightened stress reactivity	Osborne et al., 2017 [20]; Zawliska et al. 2025 [40]
Progesterone (P4)	Precursor of neurosteroids; regulates emotion	Rapid postpartum decline; sensitivity differences	Withdrawal effects in steroid-sensitive individuals	Bloch et al., 2003 [17]
Estradiol (E2)	Modulates serotonin/dopamine pathways	Heightened sensitivity to withdrawal	Limbic–prefrontal dysregulation	Barth et al., 2015 [15]; Rubinow & Schmidt, 2019 [16]
Placental CRH (pCRH)	Controls gestational timing; stress axis	Elevated or rapidly rising trajectories	Feed-forward stress activation	Yim et al., 2009 [26]; Almeida et al., 2024 [27]
Cortisol/CAR	Mediator of stress; HPA output	Altered CAR; elevated evening levels	Hyperactivation and impaired feedback	Bruce et al., 2025 [28]; Glynn & Sandman, 2014 [24]
Oxytocin	Bonding, lactation, stress buffering	Lower antepartum/postpartum levels	Impaired bonding; altered amygdala response	Skrundz et al., 2011 [31]; Feldman et al., 2010 [30]
Thyroid hormones	Regulate metabolism, serotonergic tone	Subclinical hypothyroxinemia; antibodies	Amplified vulnerability to mood dysregulation	Thompson et al., 2018 [34]; Kuijpens et al., 2001 [35]

Abbreviations. CAR: Cortisol Awakening Response; GABA-A R: Gamma-Aminobutyric Acid Type A Receptor; PPD: Peripartum Depression.

## 3. Cardiovascular and Autonomic Pathways in Peripartum Depression

Cardiovascular and autonomic adaptations are central to the physiology of pregnancy and postpartum recovery. During gestation, maternal hemodynamics undergo dramatic adjustments, including a 30–50% rise in cardiac output, reductions in systemic vascular resistance, increased plasma volume, and shifts in autonomic balance, to support fetal growth and maintain homeostasis [41]. These changes reverse after delivery, although the trajectory and efficiency of postpartum recalibration vary substantially across individuals. Emerging evidence suggests that delayed or incomplete cardiovascular and autonomic recovery may be associated with increased vulnerability to peripartum depressive symptoms, while causal relationships remain incompletely established.

A growing body of evidence indicates that women with PPD often show altered HRV, endothelial dysfunction, modified baroreflex sensitivity, increased blood pressure variability, and elevated long-term cardiovascular risk, consistent with a possible link between emotional dysregulation and cardiovascular instability [42,43]. Importantly, most of these findings derive from group-level comparisons rather than validated individual-patient thresholds. These findings situate the ANS and maternal cardiovascular system within the heart–brain axis, highlighting their importance in the multisystemic model of PPD.

Recent evidence from obstetric Doppler studies further supports the relevance of vascular regulation during complicated pregnancies. A large retrospective study by Troìa et al. examined umbilical artery pulsatility indices in pregnancies affected by gestational diabetes mellitus and reported lower umbilical artery impedance in insulin-treated women, particularly during late gestation [44]. Although focused on fetoplacental rather than maternal circulation, these findings underscore the sensitivity of vascular tone to metabolic status and therapeutic modulation during pregnancy, reinforcing the relevance of vascular markers within a broader heart–brain–endocrine framework.

### 3.1. Autonomic Nervous System: Parasympathetic Withdrawal and Sympathetic Overdrive

The ANS plays a key role in stress regulation, emotional reactivity, and adaptation to maternal demands. HRV, which reflects the dynamic interplay between sympathetic and parasympathetic branches, is one of the most robust physiological indicators of emotional regulation capacity and stress resilience [45].

Multiple studies show that women experiencing PPD have reduced HRV, particularly lower high-frequency (HF) components associated with parasympathetic (vagal) activity and reduced vagal tone [46,47]. These alterations are most consistently observed at the group level and are not yet validated as reliable individual diagnostic markers. Low HRV is also linked to impaired mood regulation, heightened amygdala reactivity, and diminished top-down cortical control, neural patterns highly consistent with PPD neurobiology [48].

Additionally, heightened sympathetic activity and blunted parasympathetic rebound have been observed postpartum in women with depressive symptoms [47]. While not specific to PPD, this autonomic profile may contribute to emotional dysregulation, reduced stress tolerance, and increased inflammatory signaling, pathways implicated in peripartum vulnerability.

### 3.2. Cardiovascular Adaptation and Hemodynamic Stress

As already outlined, pregnancy imposes substantial cardiovascular demands, elevated cardiac output, expanded blood volume, and reduced vascular resistance. While these changes are physiologic, their reversal requires coordinated neuroendocrine and autonomic recalibration.

Recent evidence indicates that women with susceptibility to PPD may show greater BPV postpartum [49], impaired endothelial function measured via flow-mediated dilation (FMD) [50], and altered arterial stiffness and pulse wave velocity (PWV), suggesting possible vascular rigidity [51]. As with HRV, these measures currently demonstrate their strongest validity at the population level, with substantial interindividual variability limiting immediate clinical applicability.

Endothelial dysfunction may be relevant because inflammatory and oxidative stress mechanisms associated with PPD can impair endothelial nitric oxide (NO) signaling, potentially contributing to interactions between mood dysregulation and vascular function [52]. These findings are consistent with the hypothesis that cardiovascular dysregulation can be viewed not only as a consequence but rather as a coexisting physiological dimension of PPD.

Evidence from fetoplacental Doppler studies further highlights the dynamic regulation of vascular resistance in metabolically complicated pregnancies. In a retrospective cohort of over 400 women with gestational diabetes, Troìa et al. reported a progressive decline in umbilical artery pulsatility index across gestation, with more pronounced reductions among women receiving insulin therapy, particularly during the third trimester [44]. While umbilical artery Doppler indices primarily reflect placental and fetal vascular resistance rather than maternal endothelial function, these data support the concept that metabolic regulation and therapeutic interventions can significantly influence vascular impedance during pregnancy. Importantly, the authors note that the prognostic value of altered umbilical artery pulsatility indices remains uncertain, underscoring limitations similar to those observed for maternal cardiovascular markers in predicting individual-level affective or clinical outcomes.

### 3.3. Baroreflex Sensitivity and Neurovisceral Integration

Baroreflex sensitivity (BRS) reflects the ability of the cardiovascular system to adjust heart rate in response to blood pressure changes. It is tightly regulated by brainstem autonomic centers, vagal afferents, and higher-order regulatory circuits, forming a central component of the neurovisceral integration model [53].

Women with PPD show reduced BRS, which correlates with blunted vagal tone and increased sympathetic activation [54]. At the group level, reduced BRS is associated with greater emotional lability, poorer stress adaptation, and diminished maternal responsiveness during the early postpartum period. This aligns with neuroimaging studies showing disrupted connectivity between the medial prefrontal cortex, insula, and amygdala, regions central to the regulation of baroreceptive and affective processes [36]. Thus, autonomic–vascular dysregulation may represent a physiological substrate for impaired affective control and maternal functioning.

### 3.4. Cardiovascular Conditions Associated with Increased PPD Risk

Several pregnancy-related cardiovascular disorders share mechanistic pathways with PPD, reinforcing the heart–brain conceptual model.

Preeclampsia is characterized by endothelial dysfunction, antiangiogenic imbalance (sFlt-1/PlGF), heightened inflammation, and autonomic dysregulation. Women with a history of preeclampsia have a twofold higher risk of postpartum depression [55]. The shared mechanisms include oxidative stress, vascular inflammation, reduced nitric oxide bioavailability, HPA-axis overactivation.

PPCM involves oxidative stress–driven cleavage of prolactin, myocardial inflammation, and angiogenic imbalance. Women with PPCM have high rates of depressive symptoms [56]. Dysregulated inflammatory and autonomic stress pathways may contribute to this overlap.

Gestational hypertension is linked to increased long-term cardiovascular disease risk and associated with heightened postpartum emotional distress [57]. These findings highlight that cardiovascular vulnerability and depressive vulnerability frequently coexist, reflecting overlapping biological pathways.

### 3.5. Long-Term Cardiovascular Risk: Maternal Heart–Brain Trajectory

Women with PPD may be at elevated risk of developing later-life cardiovascular disease, including hypertension, coronary artery disease, and stroke [58]. This observation is consistent with the possibility that PPD reflects an underlying cardiometabolic vulnerability, rather than being purely psychological. Low HRV and endothelial dysfunction, both associated with PPD (Table 2), are established predictors of long-term cardiovascular morbidity. PPD could be considered an early-life “stress test” that may reveal latent cardiovascular dysregulation.

Cardiovascular changes do not operate in isolation. They intersect with endocrine and immune mechanisms: reduced vagal tone amplifies inflammatory signaling [60]; systemic inflammation impairs endothelial function and increases sympathetic activation [39]; cortisol dysregulation modifies vascular tone and NO production. This integrative perspective supports the conceptualization of PPD as a multisystemic heart–brain–immune dysregulation syndrome, rather than a condition restricted to the CNS.

### 3.6. Clinical Feasibility and Standardization Challenges

Although cardiovascular and autonomic markers such as HRV, BPV, FMD, and BRS provide valuable insights into maternal physiological adaptation, their routine clinical implementation in peripartum care remains limited. Most evidence derives from research settings, where measures are assessed at the group level, and normative ranges for individual patients are not yet established. This limits their predictive accuracy for identifying women at risk of peripartum depression or other maternal complications.

Variability in acquisition protocols, gestational age, metabolic status, medication exposure, sleep patterns, and postpartum physiological changes further complicates standardization. For example, umbilical artery Doppler assessment, a widely used obstetric vascular measure, demonstrates detectable group-level differences in vascular impedance but limited predictive value for individual outcomes. Troìa et al. reported that insulin-treated women with gestational diabetes had lower umbilical artery pulsatility indices, especially in late gestation, yet the clinical and prognostic significance for maternal health remains uncertain [44].

Additional practical barriers include equipment availability, technical expertise, and time constraints in both obstetric and psychiatric settings. Even when technically feasible, interpretation can be confounded by physiological heterogeneity, comorbidities, and behavioral factors, such as activity, stress, and breastfeeding.

Together, these considerations highlight the need for standardized acquisition protocols, validation of normative ranges across gestational stages, and longitudinal studies to establish individual-level thresholds. Until such data are available, cardiovascular and autonomic biomarkers should be considered as research tools that complement, rather than replace, established clinical assessments in peripartum mental health care.

## 4. Immune System and Neuroinflammation in Peripartum Depression

Pregnancy is characterized by a highly dynamic and finely regulated immunological choreography that alternates between pro-inflammatory and anti-inflammatory phases. These cyclical immune shifts enable implantation, placental development, fetal tolerance, and parturition. However, disturbances in these transitions, particularly during late pregnancy and the early postpartum period, may be associated with increased vulnerability to PPD.

A substantial body of evidence suggests that neuroinflammation and immune dysregulation may mediate the relationship between physiological stress and mood instability in the peripartum period [12,39]. These processes influence both the brain and peripheral systems such as the cardiovascular and endocrine axes, reinforcing once more the multisystemic conceptualization of PPD as a heart–brain–immune disorder.

### 4.1. Immune Adaptation Across Pregnancy and Postpartum

The immune system undergoes phase-specific shifts across gestation: early pregnancy involves a pro-inflammatory environment allowing implantation and placentation; mid-pregnancy shifts toward an anti-inflammatory state that supports fetal growth; late pregnancy and labor re-engage a pro-inflammatory profile required for parturition [61].

Notably, the postpartum period is marked by immune rebound, where inflammatory markers often rise sharply. Women vulnerable to PPD may show delayed immune recalibration, potentially resulting in prolonged low-grade inflammation and neuroimmune cross-sensitization [62].

### 4.2. Peripheral Cytokines and Risk for PPD

Meta-analytic and longitudinal studies report that women who develop PPD often show elevated pro-inflammatory cytokines. The biomarkers most implicated include IL-6, TNF-α and IL-1β. IL-6 is elevated during late pregnancy and early postpartum and predicts depressive symptoms [63,64]. TNF-α is associated with anhedonia, fatigue, and sickness behavior–like symptoms [65]. IL-1β is linked to HPA-axis overactivation and depressive affect [66].

These cytokines communicate with the brain through several pathways (humoral, neural, and endothelial) modulating mood-relevant circuits. Importantly, cytokine elevation correlates not only with depressive symptoms but also with autonomic imbalance (reduced HRV) and impaired endothelial function, linking immune activation with the physiological domains described in previous chapters.

### 4.3. Microglial Activation and Central Neuroinflammation

Microglia, the resident immune cells of the CNS, play key roles in synaptic pruning, neuroplasticity, maternal behavior circuits, and threat detection. Peripartum immune challenges induce microglial priming, whereby microglia become hypersensitive to later inflammatory stimuli [67]. In vulnerable individuals, this heightened reactivity results in increased expression of pro-inflammatory cytokines within the brain, impaired neurogenesis in the hippocampus, altered connectivity in corticolimbic networks, exaggerated stress responsiveness.

Animal models of postpartum depression show intensified microglial activation in the prefrontal cortex and hippocampus during the postpartum period [68]. Translational evidence suggests similar neuroimmune signatures in humans, with increased inflammatory metabolites and glial activation markers in peripartum mood dysregulation [69].

### 4.4. Neuroimmune Crosstalk: Interaction with Stress and Neuroendocrine Systems

Inflammation interacts closely with endocrine systems central to PPD. Pro-inflammatory cytokines stimulate CRH release and increase cortisol secretion. Chronic inflammation impairs glucocorticoid receptor sensitivity, contributing to a feed-forward loop of sustained HPA-axis activation [39].

Inflammatory states reduce ALLO production and alter GABA-A receptor function, diminishing inhibitory tone and increasing stress reactivity, two hallmarks of PPD vulnerability [14]. Finally, inflammation suppresses oxytocin release and alters OXTR expression, contributing to impaired bonding, increased anxiety, and altered maternal–infant interaction [32]. These interactions illustrate how immune activation can destabilize multiple functional domains relevant to mood regulation.

### 4.5. Endothelial–Immune Interactions and Cardiovascular Pathways

Systemic inflammation directly impacts endothelial function, reducing nitric oxide availability, increasing oxidative stress, and promoting vascular stiffness. Endothelial cells themselves release cytokines and chemokines that amplify inflammatory cascades. Reduced endothelial integrity has been linked to depressive symptoms in both perinatal and non-perinatal populations [50,52].

Given the strong associations between PPD and cardiovascular dysregulation, immune–endothelial interactions provide a mechanistic pathway through which inflammation may simultaneously influence mood, vascular stability, and autonomic balance.

Preeclampsia is characterized by systemic inflammation, endothelial dysfunction, and angiogenic imbalance (sFlt-1/PlGF). Women with preeclampsia show prolonged postpartum inflammatory activation and elevated risk of depression [55].

PPCM involves myocardial inflammation, oxidative stress, and immune-driven prolactin cleavage. As already outlined, it has been observed a high prevalence of depressive symptoms in PPCM and shared inflammatory signatures [56]. Gestational diabetes is associated with chronic low-grade inflammation, oxidative stress, and increased postpartum mood disturbances. These observations are consistent with the model of PPD as involving multisystemic inflammatory vulnerability, rather than being solely a response to psychological stress.

### 4.6. Translational Implications: Immune Biomarkers and Potential Interventions

The expanding evidence linking immune dysregulation to PPD has significant translational implications, particularly for developing early risk biomarkers and novel therapeutic strategies. Several peripheral inflammatory molecules have emerged as promising predictors of vulnerability. Elevated levels of C-reactive protein (CRP), interleukin-6 (IL-6), and tumor necrosis factor-α (TNF-α) in late pregnancy and early postpartum have been repeatedly associated with depressive symptoms [63,64,65]. These cytokines are key components of low-grade systemic inflammation, which may influence HPA-axis recovery, autonomic balance, and endothelial function, three biological systems implicated in PPD.

Beyond classical cytokines, broader inflammatory metabolomic signatures are now being explored. Metabolomic analyses indicate that alterations in lipid mediators, oxidative stress–related metabolites, and immune-related biochemical pathways distinguish women who develop PPD from those who do not [70]. These approaches capture the complexity of immune activation more comprehensively than single cytokine measurements.

Particular attention has been devoted to the kynurenine/tryptophan ratio (KYN/TRP Ratio), a sensitive index of indoleamine-2,3-dioxygenase (IDO) activation. Inflammatory states upregulate IDO, diverting tryptophan metabolism away from serotonin synthesis and toward kynurenine (KYN) production. Increased KYN has been associated with depressive symptoms and stress-induced affective dysregulation in perinatal and non-perinatal populations [71]. This pathway provides a mechanistic bridge connecting inflammation, monoamine depletion, and neurotoxicity.

At the central level, neuroimmune profiling is becoming possible through the use of TSPO-targeted PET ligands, which mark microglial activation. While still experimental, early research indicates that postpartum mood disturbances may be linked to increased glial reactivity and neuroinflammation [69], aligning clinical observations with pre-clinical models demonstrating microglial sensitization during the postpartum period [67].

These insights also inform therapeutic innovation. Nutritional and pharmacological anti-inflammatory strategies, including omega-3 fatty acids, have been associated with small-to-moderate reductions in depressive symptoms, possibly through modulation of pro-inflammatory cytokines and cell membrane lipid profiles [72,73,74]. Novel cytokine-modulating agents are under investigation, although not yet tested specifically in PPD.

Another promising approach is vagal nerve stimulation (VNS), which increases parasympathetic tone and activates the “cholinergic anti-inflammatory reflex”, a pathway that dampens peripheral inflammation and improves affective regulation [60,75]. Given the observed association between reduced HRV and PPD, VNS may influence an autonomic mechanism potentially contributing to postpartum vulnerability.

Complementary mind–body therapies, such as mindfulness-based stress reduction, yoga, and slow-breathing interventions, have demonstrated reductions in inflammatory markers (e.g., IL-6, CRP) and improvements in autonomic balance [76,77]. These modalities may be particularly accessible and acceptable for postpartum women.

Finally, interventions targeting endothelial and immune homeostasis, such as structured physical activity, optimized nutrition, and sleep stabilization, have shown benefits on both inflammatory tone and depressive symptomatology [78,79]. Because endothelial dysfunction and inflammation interact bidirectionally, these lifestyle approaches may offer dual benefits for vascular and affective recovery after childbirth.

Collectively, these findings highlight the translational potential of immune-based biomarkers and therapies, supporting an integrated model of PPD that links immunology, neuroendocrinology, cardiology, and psychiatry.

Table 3 summarizes principal immune biomarkers implicated in PPD.

## 5. Heart–Brain Integration Framework

It is clear that PPD emerges not from a single dysfunctional system but from the convergence of multiple physiological domains (neuroendocrine signaling, autonomic regulation, vascular function, and immune activation) interconnected through the heart–brain axis. Viewing PPD through a heart–brain integration lens may help explain how stress, hormonal transitions, and maternal physiological adaptations jointly influence affective vulnerability.

The heart and brain engage in continuous bidirectional communication mediated by autonomic, neuroendocrine, and immune pathways. The neurovisceral integration model [59] proposes that vagally mediated cardiac flexibility reflects the functional integrity of central networks involved in emotion regulation, including the medial prefrontal cortex, anterior cingulate cortex, insula, and amygdala. During the peripartum period, these circuits undergo profound plastic changes influenced by reproductive hormones, maternal behavior circuits, and metabolic demands.

In PPD, evidence frequently indicates reductions in HRV, blunted baroreflex sensitivity, and sympathetic dominance [46,80]. These findings indicate that the central autonomic network (CAN) is disrupted, diminishing the organism’s ability to adaptively regulate physiological and emotional stress. Low vagal tone may amplify inflammatory responses and influence cardiovascular and endocrine systems, linking autonomic dysregulation to mood symptoms.

The endocrine systems, particularly ALLO, E2, P4, and placental CRH, have profound effects on autonomic regulation. ALLO enhances GABA-A receptor–mediated inhibitory tone in the limbic system, promoting parasympathetic dominance and blunting stress reactivity. When neurosteroid levels fall or receptor sensitivity is altered, this may lead to reduced parasympathetic activity, increased sympathetic arousal, and greater stress sensitivity.

Similarly, dysregulation of the HPA-axis impacts autonomic function: elevated cortisol or impaired glucocorticoid receptor sensitivity, both common in PPD, reduce vagal tone and enhance sympathetic activation [28]. These changes may influence cardiovascular dynamics, potentially contributing to endothelial dysfunction, increased arterial stiffness, and fluctuations in blood pressure variability.

Thus, neuroendocrine alterations during the peripartum period may destabilize autonomic balance, amplifying emotional vulnerability through heart–brain pathways.

### 5.1. Cardiovascular–Neuroendocrine–Inflammatory Triad

Cardiovascular physiology plays an active, not incidental, role in emotional well-being. Reduced endothelial function, decreased FMD, and increased arterial stiffness (PWV) have been observed in women with postpartum mood disturbances [50]. These vascular alterations are closely linked to systemic inflammation, which impairs nitric oxide bioavailability, increases oxidative stress, and activates endothelial cytokine signaling.

Inflammatory activation also interacts with neuroendocrine systems: cytokines stimulate CRH release, alter serotonin metabolism (via IDO activation), and prime microglia, contributing to neuroinflammation [39,67]. Low vagal tone further amplifies these immune responses, creating a positive feedback loop in which cardiovascular instability, neuroendocrine shifts, and immune dysregulation reinforce each other.

This vascular–immune–neuroendocrine triad may represent a physiological substrate through which stress and environmental challenges contribute to PPD vulnerability.

### 5.2. Maternal Brain Plasticity and Heart–Brain Dynamics

The postpartum period is marked by profound remodeling of neural circuits involved in attachment, threat detection, reward processing, and stress adaptation [81]. Maternal brain plasticity, driven by hormonal, sensory, and behavioral input, optimizes caregiving but also increases vulnerability to stress and inflammation.

Functional neuroimaging studies show that women with PPD exhibit increased amygdala reactivity to infant cues, decreased prefrontal regulation, reduced reward response in striatal regions, altered intrinsic connectivity within default mode and salience networks [36].

These neural changes correlate with HRV reductions and inflammatory markers, indicating that heart–brain and brain–immune pathways are synchronized during the peripartum period. When these processes are dysregulated, emotional stability and maternal sensitivity may be affected.

Heart–brain integration allows rapid adaptation to physiological and emotional challenges. However, when multiple regulatory systems (autonomic, cardiovascular, endocrine, and immune) experience simultaneous dysregulation, resilience capacity diminishes. Factors influencing vulnerability include genetic and epigenetic sensitivity to hormonal shifts [18], history of childhood adversity affecting immune and autonomic regulation [38], cardiometabolic predisposition (e.g., preeclampsia, PPCM), and persistent inflammatory activation.

Conversely, protective mechanisms include robust vagal tone, balanced endocrine response, preserved endothelial function, and strong maternal social support networks.

### 5.3. A Unified Heart–Brain Model for PPD

The heart–brain integration perspective conceptualizes PPD as involving neuroendocrine sensitivity to hormone fluctuations, autonomic dysregulation (low HRV, reduced BRS, sympathetic predominance), vascular instability (endothelial dysfunction, altered PWV/FMD), immune activation and neuroinflammation, and alterations in maternal brain circuitry.

These components interact dynamically through bidirectional pathways: inflammation impairs endothelial and neural function; autonomic imbalance amplifies inflammatory tone; endocrine shifts alter autonomic and cardiovascular regulation; cardiovascular dysregulation feeds back on mood circuits.

**Figure 3:**
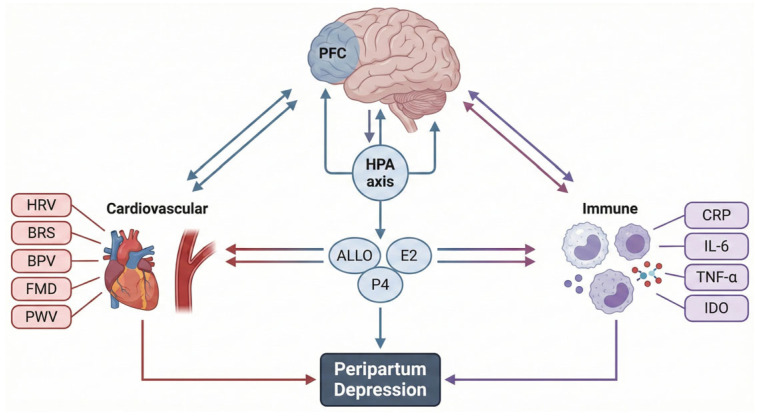
Integrated Heart–Brain–Endocrine–Immune Model of Peripartum Depression. Abbreviations. PFC: Prefrontal Cortex; HRV: Heart Rate Variability; BRS: Baroreflex Sensitivity; HPA axis: Hypothalamic–Pituitary–Adrenal Axi; ALLO: Allopregnanolone; E2: Estradiol; P4: Progesterone; BPV: Blood Pressure Variability; FMD: Flow-Mediated Dilation; PWV: Pulse Wave Velocity; CRP: C-reactive Protein; IL-6: Interleukin-6; TNF-α: Tumor Necrosis Factor-alpha; IDO: Indoleamine-2,3-dioxygenase.

This integrative model positions PPD as a multisystemic heart–brain–immune syndrome, offering a biologically grounded framework to guide prevention, diagnosis, and intervention. To illustrate the multisystemic nature of peripartum depression, Figure 3 provides a conceptual heart–brain integration model summarizing the dynamic interactions among neural circuits, autonomic regulation, endocrine transitions, cardiovascular physiology, and immune activation. This framework highlights how dysregulation across these interconnected systems can converge to increase maternal vulnerability during the peripartum period.

## 6. Biomarkers and Clinical Translation

The increasing recognition of PPD as a multisystemic disorder involving neural, endocrine, autonomic, cardiovascular, and immune pathways has accelerated interest in identifying biomarkers that can guide risk prediction, early detection, and personalized intervention. While no single biomarker provides definitive diagnostic precision, converging evidence indicates that integrated biomarker panels, reflecting heart–brain dynamics, may hold promise for clinical translation. This chapter reviews the most robust candidate biomarkers and outlines how they may contribute to precision medicine approaches in peripartum mental health.

Hormonal fluctuations represent one of the earliest and most biologically grounded indicators of PPD vulnerability. Sharp declines in E2 and P4 immediately postpartum, together with individual differences in neural sensitivity to these hormones, may contribute to affective instability. Low levels or impaired receptor sensitivity to ALLO, a potent GABA-A receptor modulator, have been associated with increased stress reactivity and depressive symptoms [14].

Activity of the HPA axis, reflected in metrics such as CAR and diurnal cortisol slope, predicts both antenatal anxiety and postpartum depressive episodes [29]. Elevated CRH in late pregnancy has consistently predicted PPD in longitudinal studies, supporting its potential role as a high-specificity endocrine marker.

Given their interdependency, combinations of neurosteroid profiles and HPA-axis measures may provide a more reliable biomarker signature than individual assays.

Autonomic markers reflect real-time physiological adaptation and may detect vulnerability even before mood symptoms appear. High-frequency HRV (HF-HRV), RMSSD (Root Mean Square of Successive Differences), and BRS provide non-invasive measures of vagal function. Reduced HRV, frequently reported in PPD research, is associated with impaired emotion regulation, increased amygdala reactivity, and inflammatory amplification [59,79].

Early postpartum elevations in BPV further indicate autonomic–vascular dysregulation and have been associated with anxiety, sleep disturbances, and mood reactivity [49].

Wearable technologies have made continuous HRV monitoring feasible, raising the possibility of scalable screening tools for real-world risk assessment.

The cardiovascular system provides structural and functional metrics that mirror autonomic and inflammatory changes. FMD and pulse wave velocity (PWV) reflect endothelial health and arterial stiffness, both of which are influenced by inflammation, oxidative stress, and hormonal transitions.

Reduced FMD and increased PWV have been observed in women with postpartum depressive symptoms, suggesting a potential link between vascular dysregulation and affective symptoms [50]. Additional markers, such as arterial elastance and microvascular reactivity, may further refine cardiovascular risk profiling in peripartum mental health.

Inflammatory biomarkers, including CRP, IL-6, TNF-α, IL-1β, and cytokine composite panels, are among the most consistently associated with PPD. These molecules modulate neural circuits via effects on serotonin metabolism, HPA-axis activation, and microglial priming [39]. The KYN/TRP Ratio, reflecting IDO activity, has emerged as a highly promising metabolic biomarker linking peripheral inflammation to central monoamine depletion and neurotoxicity [71]. Emerging neuroimaging modalities, such as TSPO PET, may allow direct quantification of microglial activation in future clinical studies.

Multi-marker immune profiles, rather than single cytokines, may provide greater specificity and predictive value.

Neuroimaging studies have identified functional and structural patterns associated with PPD, including heightened amygdala reactivity to infant cues, reduced prefrontal cortex (PFC) regulation, decreased ventral striatal reward signaling, altered connectivity within the default mode and salience networks [36].

Although not currently suitable for routine screening, neuroimaging biomarkers may offer mechanistic insights and help identify biological subtypes of PPD, potentially guiding targeted interventions.

Given PPD’s multisystemic physiology, integrated multimodal biomarker panels offer the highest translational potential. Combining endocrine, autonomic, immune, and vascular markers with clinical and psychosocial risk factors increases predictive accuracy compared to any single metric. Examples of clinically promising integrated approaches include HPA-axis dysregulation with HRV reduction, CRP/IL-6/TNF-α panel with endothelial dysfunction, KYN/TRP Ratio with autonomic imbalance, neurosteroid levels with functional connectivity changes.

Machine learning models trained on multimodal biomarker datasets have begun to show strong predictive capabilities for peripartum mood outcomes [82].

While integrated biomarker panels hold promise for risk stratification and early detection of PPD, several practical considerations must be addressed before routine clinical adoption. First, standardization of sampling protocols, assay platforms, and timing across gestational and postpartum periods is essential to ensure reproducibility and comparability of results. Second, the accessibility and cost-effectiveness of multimodal assessments, including neuroimaging, HRV monitoring, and multiplex cytokine panels, may limit scalability in routine obstetric practice. Third, interpretation of biomarker data requires integration with clinical and psychosocial risk factors, highlighting the need for validated predictive algorithms and decision-support tools. Finally, ethical considerations, patient acceptability, and feasibility of longitudinal monitoring should guide implementation strategies. Addressing these practical challenges will be critical to translating the multisystemic biomarker framework into actionable precision perinatal psychiatry.

Nevertheless, the convergence of systems biology, neuroendocrinology, and digital phenotyping suggests the potential for future integration of biomarker-guided screening into perinatal care.

## 7. Therapeutic Implications and Future Horizons

The heart–brain–endocrine–immune perspective on PPD not only expands our understanding of its pathophysiology but also reshapes therapeutic priorities. Traditional treatments for PPD have mainly targeted mood symptoms, often without addressing the multisystemic disturbances that may precede or accompany the disorder. Emerging evidence suggests that interventions capable of modulating neuroendocrine, autonomic, cardiovascular, and immune pathways may offer more comprehensive and durable benefits. Within this framework, it is important to distinguish interventions with established efficacy from adjunctive strategies and those that remain experimental or hypothetical, particularly when evidence is derived from non-peripartum populations or indirect studies.

### 7.1. Neuroendocrine-Based Treatments

Hormonal modulation represents one of the most transformative developments in PPD treatment. The approval of brexanolone and zuranolone, neuroactive steroid formulations acting as positive allosteric modulators of the GABA-A receptor, underscores the centrality of neurosteroid signaling in postpartum affective regulation. These therapies are considered established treatments, demonstrating rapid restoration of inhibitory tone, reduction in stress reactivity, and improvement in mood within hours to days [40,83].

Beyond neurosteroids, interventions targeting HPA-axis dysregulation are under investigation. CRH antagonists, cortisol synthesis inhibitors, and modulators of glucocorticoid receptor sensitivity represent promising avenues for individuals with pronounced endocrine vulnerability, although most remain experimental.

### 7.2. Autonomic Modulation and Neuromodulation Therapies

Given the consistent association between reduced HRV, heightened sympathetic drive, and PPD, interventions targeting the ANS are gaining traction.

Vagal Nerve Stimulation (VNS) enhances parasympathetic tone, activates the cholinergic anti-inflammatory reflex, and has shown antidepressant effects in treatment-resistant depression [84]. Its mechanistic overlap with autonomic and immune pathways implicated in PPD supports its potential use in postpartum application.

Transcutaneous Auricular VNS (taVNS) is a non-invasive technique using electrodes on the outer ear (auricle) to stimulate the auricular branch of the vagus nerve. It is a safer, wearable-friendly approaches appropriate for postpartum women. Early trials indicate improvements in HRV, mood regulation, and inflammatory profiles [85].

HRV Biofeedback trains individuals to increase vagal tone through paced breathing and autonomic awareness represents an adjunctive strategy in peripartum women. Evidence indicates reductions in anxiety, improved stress tolerance, and better emotional regulation [86], suggesting its suitability as a low-cost adjunctive therapy suitable for perinatal care.

Targeting the dorsolateral prefrontal cortex, repetitive Transcranial Magnetic Stimulation (rTMS) improves mood and executive control, and may normalize autonomic patterns through top-down regulation of the central autonomic network. Peripartum-specific trials remain limited but promising [87].

### 7.3. Anti-Inflammatory and Immunomodulatory Approaches

Recognition of neuroinflammation as a core risk factor for PPD has encouraged interest in anti-inflammatory interventions.

Long-chain omega-3 polyunsaturated fatty acids (EPA and DHA) are considered established adjunctive treatments, reducing IL-6, CRP, and TNF-α levels and have shown moderate antidepressant effects in perinatal women, particularly those with elevated inflammatory markers [72,74].

Dietary patterns rich in polyphenols, antioxidants, fiber, and omega-3s (e.g., Mediterranean diet) represent adjunctive strategies capable of modulating both systemic inflammation and endothelial function, offering potential synergistic benefits for mood and cardiovascular stability [88].

Pharmacologic inhibitors of IDO, antioxidants, and agents targeting KYN metabolism may reduce neurotoxic metabolite production and restore monoamine balance. Although early-phase research shows promise, clinical trials specifically in PPD remain limited.

Regular moderate exercise is an established adjunctive intervention, reducing inflammatory tone, increases HRV, improving endothelial function, and enhancing neurotrophic signaling [78]. Exercise is among the most accessible and globally recommended first-line interventions for postpartum well-being.

### 7.4. Cardiometabolic and Endothelial-Targeted Interventions

Because endothelial dysfunction and vascular instability contribute to PPD risk, strategies that improve vascular health may indirectly support mood recovery. Aerobic exercise improves FMD, reduces PWV, and enhances nitric oxide signaling; antioxidant supplements (e.g., vitamins C and E, L-arginine) may support endothelial repair; management of hypertensive disorders (e.g., preeclampsia) is crucial to prevent long-term cardiovascular and mood-related morbidity; early postpartum BPV monitoring represents an adjunctive strategy for targeting autonomic and lifestyle interventions.

This vascular perspective broadens PPD treatment beyond psychotropic agents and underscores the importance of interdisciplinary obstetric–cardiology–psychiatry collaboration.

### 7.5. Mind–Body and Behavioral Interventions

Mind–body therapies constitute adjunctive strategies and influence multiple biological pathways central to PPD vulnerability.

Mindfulness-Based Interventions reduce inflammatory markers, modulate HPA reactivity, increase HRV, and improve emotional regulation [76]. Particularly useful in women with high stress loads or traumatic birth experiences. At the same way, yoga and breathing-based practices promote parasympathetic activation, improve sleep, reduce anxiety, and enhance autonomic flexibility [89].

Cognitive Behavioral Therapy (CBT) and Interpersonal Therapy (IPT) remain gold-standard psychotherapies, with robust efficacy for postpartum mood disorders. When combined with biological interventions (e.g., neurosteroid therapy or HRV-biofeedback), synergistic effects may emerge [90].

### 7.6. Digital Health, Wearables, and Precision Psychiatry

Wearables capable of monitoring HRV, sleep patterns, activity levels, and BPV are emerging as powerful tools for continuous risk assessment. Digital phenotyping can identify deviations in autonomic function or stress responsivity days or weeks before clinical symptoms emerge [91].

Machine learning platforms integrating biomarker, behavioral, and psychosocial data could support early-warning systems, personalized treatment plans, and real-time monitoring of therapeutic response.

### 7.7. Future Horizons: Toward a Multisystem Therapeutic Model

Looking ahead, the most promising therapeutic strategies will likely address multiple biological domains simultaneously. Potential directions include combined neurosteroid + HRV-biofeedback protocols, VNS with anti-inflammatory nutraceuticals, personalized biomarker-guided treatment algorithms, cardiovascular risk monitoring integrated with perinatal psychiatric care, interventions targeting microglial priming during pregnancy or postpartum.

**Figure 4:**
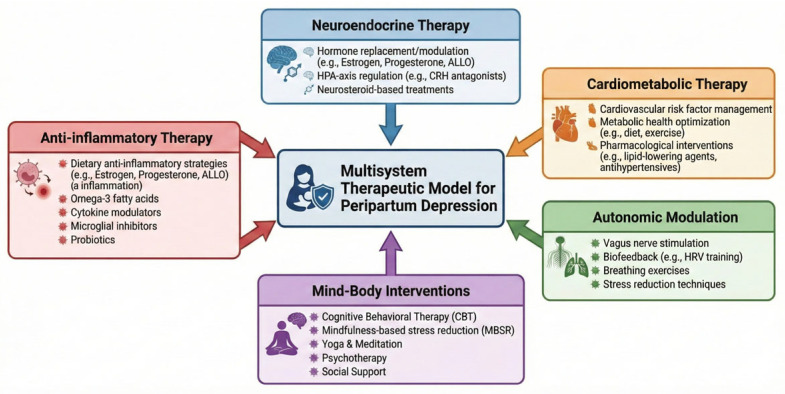
Multisystem Therapeutic Model for Peripartum Depression.

Integration of cardiovascular, neuroendocrine, autonomic, and immune perspectives frames PPD as a multisystem heart–brain disorder, with implications for earlier detection, broader therapeutic targets, and potentially improved prevention. Figure 4 presents an integrated multisystem therapeutic model. This conceptual illustration highlights five interconnected domains, neuroendocrine therapy, autonomic modulation, anti-inflammatory interventions, mind–body approaches, and cardiometabolic therapy, each targeting specific physiological mechanisms implicated in peripartum depression. The circular organization emphasizes that these systems are dynamically linked, and that effective prevention and treatment strategies may require simultaneous modulation across multiple biological pathways rather than a single-system approach.

## 8. Limitations

Despite notable progress, the current literature on the multisystem biology of PPD shows several limitations. First of all, most studies tend to adopt a single-system perspective, examining endocrine, immune, autonomic or neural pathways in isolation rather than simultaneously [12,39]. This reductionist approach may limit the identification of reproducible biomarkers that capture dynamic heart–brain interactions. Major biomarkers, such as cytokines, neurosteroids and HRV, exhibit methodological heterogeneity, including variations in timing, assay platforms and analytical approaches [64,70]. Additionally, many cohorts include small, demographically homogeneous samples, limiting generalizability across diverse ethnic and socioeconomic populations [63]. Longitudinal, multimodal studies remain scarce, limiting insight into temporal progression from biological dysregulation during pregnancy to postpartum symptom onset [92]. Very few studies integrate biological, clinical and psychosocial data within a unified analytic framework [49]. Interventional trials addressing inflammation, autonomic imbalance or neuroendocrine sensitivity remain limited, and mechanistic endpoints demonstrating causal pathways are often lacking [93].

Future research should consider integrative, multimodal frameworks to better capture the complexity of PPD. Large cohorts collecting endocrine, autonomic, cardiovascular, immune and neuroimaging markers alongside clinical and psychosocial variables are needed to delineate biological trajectories and risk phenotypes [94]. Machine-learning approaches may help derive personalized risk models and identify biomarker-defined subtypes [82]. The growing availability of digital phenotyping and wearable HRV/BPV monitoring offers opportunities for real-time early-warning systems [91]. Mechanistic clinical trials should evaluate whether interventions targeting neurosteroid deficits [83], autonomic imbalance [86], inflammation [74] or endothelial dysfunction [50] produce measurable physiological improvements consistent with heart–brain integration models. Additional priorities include establishing pregnancy- and postpartum-specific reference ranges [66], expanding studies to diverse populations, advancing microglial imaging [69], and integrating cardiology and psychiatry within perinatal care. Ultimately, future research may facilitate the development of multisystem therapeutic algorithms guided by biomarker signatures.

## 9. Conclusions

PPD can be conceptualized as a prototypical heart–brain disorder in which neuroendocrine transitions, autonomic imbalance, vascular instability and immune activation may converge to influence maternal vulnerability [40,59]. Throughout pregnancy and the postpartum period, hormonal withdrawal, reduced vagal tone, endothelial dysfunction and microglial sensitization interact dynamically, creating a multisystem context for affective dysregulation [67,95]. Interventions targeting a single-domain may be insufficient. Multimodal therapeutic strategies, combining neurosteroid replacement [40], autonomic modulation [93], anti-inflammatory approaches [72], mind–body therapies [76] and cardiometabolic optimization, may offer enhanced potential for prevention and long-term stabilization. As the field moves toward precision perinatal psychiatry, biomarker-guided screening, digital monitoring and integrated therapeutics may transform early detection and personalized care [82]. Conceptualizing PPD through a heart–brain integration lens provides a biologically coherent and potentially clinically actionable framework for advancing maternal mental health.

## Figures and Tables

**Table 2:** Cardiovascular and Autonomic Biomarkers Associated with Peripartum Depression.

Biomarker	Physiological Role	Pattern Associated with PPD Risk	Mechanistic Implications	Key References
HRV	Index of autonomic flexibility; vagal tone; stress regulation	Reduced HF-HRV; increased sympathetic dominance	Impaired emotion regulation; increased amygdala reactivity	Brandes-Aitken et al., 2024 [46]; Shinba et al., 2024 [47]; Thayer et al., 2012 [48]
BRS	Reflex control of heart rate and BP; neurovisceral integration	Reduced postpartum BRS	Poor autonomic–cardiovascular coupling; heightened stress sensitivity	Shah et al., 2020 [54]; Thayer & Lane, 2009 [59]
PWV	Measure of arterial stiffness; vascular aging	Increased PWV postpartum, particularly after hypertensive disorders	Endothelial dysfunction; increased cardiovascular risk	Lee et al., 2025 [51]
FMD	Endothelial NO-dependent vasodilation	Reduced postpartum FMD in women with depressive symptoms or preeclampsia	Endothelial dysfunction; inflammation and oxidative stress	Ackerman-Banks et al., 2023 [50]
BPV	Marker of autonomic–vascular regulation	Increased early postpartum BPV	Sympathetic dominance; reduced vascular stability	Wu et al., 2024 [49]
RMSSD	Parasympathetic HRV component; beat-to-beat variability	Lower RMSSD in PPD	Reduced vagal tone; impaired stress buffering	Shaffer & Ginsberg, 2017 [45]
LF/HF Ratio	Balance between sympathetic and parasympathetic drive	Elevated LF/HF in postpartum depressive symptoms	Sympathetic overactivation	Shinba et al., 2024 [47]

Abbreviations. HRV: Heart Rate Variability; HF-HRV: High-Frequency Heart Rate Variability (parasympathetic/vagal component); BRS: Baroreflex Sensitivity; PWV: Pulse Wave Velocity; FMD: Flow-Mediated Dilation; BPV: Blood Pressure Variability; RMSSD: Root Mean Square of Successive Differences (parasympathetic HRV index); LF/HF Ratio: Low-Frequency/High-Frequency Ratio (autonomic balance indicator); NO: Nitric Oxide; PPD: Peripartum Depression; BP: Blood Pressure.

**Table 3:** Immune Biomarkers Implicated in Peripartum Depression.

Biomarker	Physiological Role	Pattern Associated with PPD Risk	Mechanistic Implications	Key References
CRP	Acute-phase protein; systemic inflammation marker	Elevated CRP in late pregnancy and early postpartum	Promotes systemic inflammatory tone; interacts with HPA-axis and endothelial dysfunction	Skalkidou et al., 2012 [63]; Li et al., 2022 [70]
IL-6	Pro-inflammatory cytokine; modulates stress pathways	Increased IL-6 during late pregnancy predicts PPD; elevated postpartum	Stimulates CRH release; alters serotonin metabolism; enhances microglial activation	Christian et al., 2009 [66]; Kurniati et al., 2025 [64]
TNF-α	Key mediator of inflammation and sickness behavior	Higher TNF-α postpartum in women with depressive symptoms	Induces IDO activation; increases oxidative stress; disrupts neuroplasticity	Corwin et al., 2008 [65]; Dye et al., 2022 [62]
IL-1β	Pro-inflammatory cytokine involved in HPA activation	Elevated IL-1β in pregnancy linked to later depressive symptoms	Enhances CRH secretion; contributes to neuroinflammation and altered stress reactivity	Christian et al., 2009 [66]; Payne et al., 2019 [67]
KYN/TRP Ratio	Reflects IDO activity and immune-induced serotonin metabolism	Increased KYN/TRP Ratio in inflammatory states associated with depressive symptoms	Serotonin depletion; neuroactive metabolite production	Savitz, 2020 [71]; Li et al., 2022 [70]
TSPO PET Ligands	Marker of microglial activation in the CNS	Preliminary evidence of increased TSPO binding in postpartum mood disturbances	Indicates central neuroinflammation; microglial priming	Bollinger et al., 2021 [69]; Payne et al., 2019 [67]
Cytokine Panels	Integrative profiling of immune function	Combined IL-6/CRP/TNF-α signatures predict higher PPD risk	Multidimensional immune dysregulation; interaction with HPA-axis and ANS	Osborne & Monk, 2013 [12]; Dye et al., 2022 [62]

Abbreviations. CRP: C-reactive protein; IL-6: Interleukin-6; TNF-α: Tumor Necrosis Factor-alpha; IL-1β: Interleukin-1 Beta; KYN/TRP Ratio: Kynurenine/Tryptophan Ratio; IDO: Indoleamine-2,3-dioxygenase; TSPO: Translocator Protein (18 kDa), marker of microglial activation; PET: Positron Emission Tomography; CNS: Central Nervous System; ANS: Autonomic Nervous System.

## Data Availability

No new data were created or analyzed in this study.

## Abbreviations

The following abbreviations are used in this manuscript:

ALLO	Allopregnanolone
ANS	Autonomic Nervous System
BPV	Blood Pressure Variability
BRS	Baroreflex Sensitivity
CAR	Cortisol Awakening Response
CRH	Corticotropin-Releasing Hormone
E2	Estradiol
FMD	Flow-Mediated Dilation
HPA axis	Hypothalamic–Pituitary–Adrenal Axis
HRV	Heart Rate Variability
IDO	Indoleamine-2,3-Dioxygenase
IL-6	Interleukin-6
KYN	Kynurenine
KYN/TRP Ratio	Kynurenine-to-Tryptophan Ratio
P4	Progesterone
PFC	Prefrontal Cortex
PPD	Peripartum Depression
PWV	Pulse Wave Velocity
RMSSD	Root Mean Square of Successive Differences
TNF-α	Tumor Necrosis Factor-alpha
TSPO	Translocator Protein
VNS/taVNS	(Transcutaneous) Vagus Nerve Stimulation
